# Comprehensive assessment of the association between estrogen receptor of alpha polymorphisms and the risk of prostate cancer: evidence from a meta-analysis

**DOI:** 10.18632/oncotarget.21117

**Published:** 2017-09-21

**Authors:** Guang Li, Meng Yang, Xian Li, Shixiong Deng

**Affiliations:** ^1^ Laboratory of Forensic Medicine and Bioinformatics, Chongqing Medical University, Chongqing, China; ^2^ Research Department, Children Hospital of Chongqing Medical University, Chongqing, China; ^3^ Laboratory of Biomedical Engineering, Chongqing Medical University, Chongqing, China

**Keywords:** estrogen receptor of alpha, polymorphisms, prostate cancer, meta-analysis

## Abstract

We performed a meta analysis to access the relationship of estrogen receptor of alpha (ESRα) polymorphisms with the risk of prostate cancer (PC). Twenty-four case-control studies (including 5477 cases and 10708 controls) were recruited for meta-analysis. The strongest association with the risk of PC was observed between ESRα rs9340799 and rs2234693 under the two genotypic models of allele and codominance in the overall population (*p* < 0.05). Under the subgroup analysis of ethnicity, we observed that ESRα rs9340799 was significantly associated with the susceptibility to PC in European population (AvsG, *p* = 0.000; AAvsGG, *p* = 0.002), while there was no difference in Asian (AvsG, *p* = 0.493; AAvsGG, *p* = 0.736) or African population (AvsG, *p* = 0.800; AAvsGG, *p* = 0.788). The results also showed that significant association between rs2234693 and the susceptibility to PC in European (CvsT, *p* = 0.004; CCvsTT, *p* = 0.001) and Asian population (CvsT, *p* = 0.004; CCvsTT, *p* = 0.003), but not in African population (CvsT, *p* = 0.636; CCvsTT, *p* = 0.669). The meta-analysis indicated that ESRα rs9340799 and rs2234693 might contribute to susceptibility and development of PC in European population.

## INTRODUCTION

Prostate cancer (PC) is a common malignant tumor occurring in males and has become the predominant cause of death among males, which accounts for about 10% of male mortality [1]. However, the pathogenesis of PC remains to be determined. Several studies reported that both genetic susceptibility and environmental factors such as diet and lifestyle exerted major influences on the occurrence of PC [2]. In particular, single nucleotide polymorphism (SNP) derived from single nucleotide point mutation was considered to be one of the important material carriers with genetic susceptibility [3]. As the third generation genetic marker, SNP was polymorphic and derived from the variation of individual nucleotide at the same site of DNA sequence. Due to the diversity in diet, geological areas and genetic factors, the incidences of PC among races remained differences and similarities. Estrogen receptor of alpha (ESRα), found by Jensen in 1971, was an estrogen-dependent transcription factor, and located at the Zone 1, Area 25 of long-arm of the No. 6 chromosome (6q25.1), and the polymorphic sites of its intron and exon areas had been already identified [4]. Meanwhile, as a member of nucleus receptor super-family, ESRα could transfer into nucleus of PC cells in combination with estrogen, regulate the signal pathways of gene transcription and promote cell proliferation [5]. Currently, A number of anti-cancer drugs play important roles in disturbing and hindering the combination of the nodes to achieve the therapeutic goal [6]. It is possible that the development of PC may be affected by the SNP site of ESRα. However, the results were not always consistent [9–12]. Different regional and ethnic groups varied from inherit susceptibility genes and SNPs due to the highly genetic heterogeneity of PC.

Although there are several studies on investigating the interactions between ESRα and PC among Asians, Europeans and Africans [7, 8], the inconsistency of findings exists. Remarkably, both sites of rs9340799 and rs2234693 have been widely studied in ESRα genetic polymorphisms [9–12], but ESRα rs9340799 or rs2234693 were independent of PC susceptibility because of quality and quantity limitations. The meta-analysis of ESRα rs9340799 and rs2234693 was firstly conducted by Ding [10] in 2012, indicating that rs9340799, not rs2234693, confered an elevated risk of PC. Gu's study [11] demonstrated that there was no significant difference between 4,884 case groups and 10,134 control groups in rs2234693 regardless of allele or other models (allele model, OR = 0.98; 95 % CI, 0.88–1.08; *P* = 0.685; dominant model, OR = 0.98; 95 % CI, 0.89–1.07; *P* = 0.685; recessive model, OR = 0.96; 95 % CI, 0.81–1.14; *P* = 0.657; homozygous model, OR = 0.96; 95 % CI, 0.77–1.19; *P* = 0.708; heterozygous model, OR = 0.96; 95 % CI, 0.81–1.13; *P* = 0.708, respectively). On the other hand, G in rs9340799 may be related to PC susceptibility (allele model, OR= 1.09; 95 % CI, 1.03–1.17; *P* = 0.006; dominant model, OR = 1.17; 95 % CI, 1.07–1.29; *P* = 0.001; homozygous model, OR = 1.17; 95 % CI, 1.01–1.35; *P* = 0.040; respectively), especially in Africans (allele model, OR = 1.53; 95 % CI, 1.13–2.07; *P* = 0.006; dominant model, OR = 1.78; 95 % CI, 1.19–2.66; *P* = 0.005; homozygous model, OR = 2.04; 95 % CI, 1.00–4.14; *P* = 0.049, respectively). In contrast, such connections had not been indentified yet in Europeans or Asians, as reported in Ding's publications. Fu's research[9] presented that rs9340799 polymorphism was significantly associated with PC in overall populations (GG+GA vs. AA: P= 0.002; G vs. A: *P* = 0.004), Caucasians (GG+GA vs. AA: *P* = 0.008; G vs. A: *P* = 0.016) and Africans (GG+GA vs. AA: *P*= 0.005; G vs. A: *P* = 0.006), but not in Asians (GG+GA vs. AA: *P* = 0.462; G vs. A: *P* = 0.665). Another study [12] regarding 4,623 cases of PC patients pointed out that rs2234693 in overall populations was markedly involved in PC susceptibility (*P* < 0.05), and the polymorphism CC may increase the risks of PC. Moreover, ethnic subgroup analysis revealed that the site had a close correlation with Europeans (*P* < 0.05), but not with Asians or Africans (*P* > 0.05).

It has been shown that there is certain correlation between PC susceptibility and ESRα rs9340799 or rs2234693, but unanimous conclusion has not been reached yet in the repetitive studies. Therefore, it remains to be verified further. This study aims to assess the association of ESRα sites rs9340799, rs2234693 and the susceptibility of PC according to all published literatures using Cochrane system assessment method. These findings will provide reliable evidence for therapeutic approahes in PC disease.

## RESULTS

### Studies included in the meta-analysis

**Figure 1:**
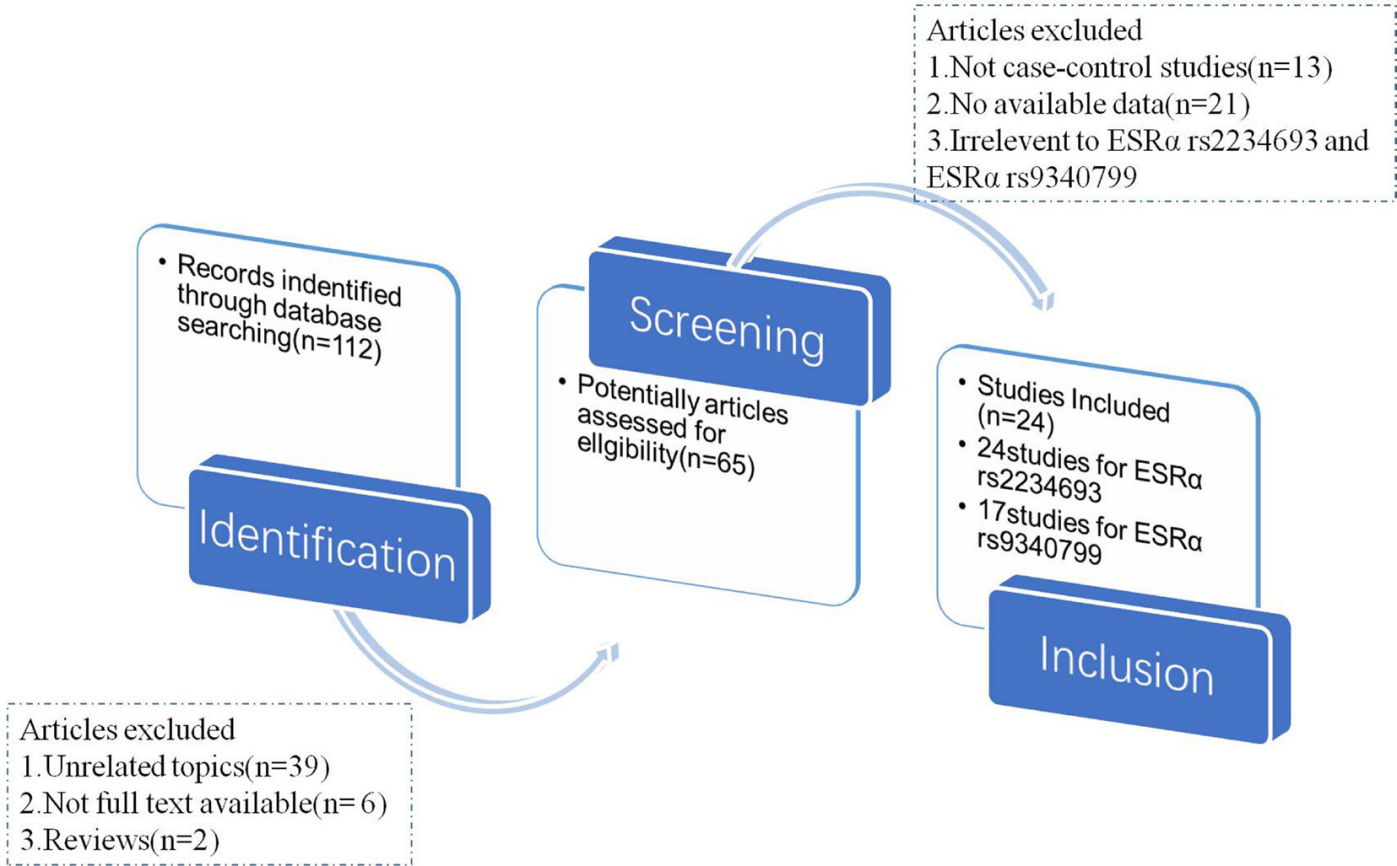
The flow chart of article selected in meta-analysis

In the meta-analysis, totally 112 relevant articles were obtained under some fundamental restrictions. After filtering, 24 eligible articles [14–37] were finally selected on the basis of the inclusion and exclusion criteria. The flow chart of selecting articles process was presented in Figure 1. Therefore, there were 41 independent case-control studies in total, and the genotype distribution of control group was based on Handy-Weinberg. For ESRα rs9340799, there were 17 studies involving a total of 3960 PC cases and 4848 normal controls. For ESRα rs2234693, 24 studies (5477 PC cases and 10708 normal controls) were available, respectively. The main characteristics of these included studies were shown in Table 1.

**Table 1:** The main characteristics of all eligible studies in meta-analysis

	Study		Sample size (case/control)		Genotype Distribution (case)			Genotype Distribution (control)			Ethnicity	HWE *p*-value
	ESRα rs9340799 (A>G)				AA	AG	GG	AA	AG	GG		
1	Modugno	2001	82	237	34	38	10	116	93	28	Caucasian	0.175
2	Suzuki	2003	101	114	72	24	5	75	30	9	Asian	0.147
3	Fukatsu	2004	117	242	74	37	6	163	68	11	Asian	0.286
4	Hernandez(a)	2006	120	303	56	51	13	153	119	31	Caucasian	0.274
5	Hernandez(b)	2006	431	582	182	191	58	229	281	72	Caucasian	0.371
6	Hernandez(c)	2006	47	213	17	25	5	117	77	19	African	0.226
7	Cunningham	2007	918	487	370	417	121	189	227	71	Caucasian	0.847
8	Beuten(a)	2009	82	209	37	36	9	118	78	13	African	1
9	Beuten(b)	2009	195	371	91	84	20	224	88	59	Caucasian	1
10	Beuten(c)	2009	609	843	258	277	74	335	393	115	Caucasian	1
11	Gupta	2010	157	170	71	75	11	87	72	11	Asian	0.565
12	Sissung	2010	129	127	42	69	18	58	61	8	Caucasian	0.146
13	Balistreri	2011	50	47	34	13	3	42	4	1	Caucasian	0.156
14	Szendroi	2011	205	101	35	111	59	29	54	18	Caucasian	0.545
15	Safarinejad	2012	162	324	20	108	34	81	187	56	Caucasian	0.05
16	Jurecekova	2013	311	256	110	145	56	119	105	32	Caucasian	0.259
17	Han	2017	244	222	148	84	12	142	70	10	Asian	0.68
	**ESRα rs2234693 (C>T)**				**CC**	**CT**	**TT**	**CC**	**CT**	**TT**		
1	Modugno	2001	81	237	21	34	26	43	109	85	Caucasian	0.424
2	Tanaka	2003	115	200	29	63	23	48	113	39	Asian	0.088
3	Suzuki	2003	101	114	12	43	46	26	59	29	Asian	0.851
4	Fukatsu	2004	116	238	22	57	37	47	110	81	Asian	0.427
5	Hernandez(a)	2006	120	303	18	55	47	43	131	129	Caucasian	0.318
6	Hernandez(b)	2006	431	582	100	216	115	132	296	154	Caucasian	0.679
7	Hernandez(c)	2006	47	213	16	22	9	50	113	50	African	0.413
8	Low	2006	75	158	21	41	13	25	84	49	Caucasian	0.329
9	Cunningham	2007	924	489	213	454	257	120	249	120	Caucasian	0.718
10	Berndt	2007	470	603	111	238	121	135	316	152	Caucasian	0.254
11	Kjaergaard	2007	116	4005	26	55	35	830	1972	1203	Caucasian	0.676
12	Sobti	2008	157	170	52	77	28	64	90	16	Asian	1
13	Onsory	2008	100	100	18	54	28	10	48	42	Asian	0.656
14	Beuten(a)	2009	82	209	23	41	18	50	105	54	African	1
15	Beuten(b)	2009	195	514	28	92	75	82	246	186	Caucasian	1
16	Beuten(c)	2009	609	843	138	304	167	200	421	222	Caucasian	1
17	Gupta	2010	157	170	28	77	52	16	90	64	Asian	0.066
18	Sonoda	2010	180	177	31	89	60	29	87	61	Asian	0.878
19	Sissung	2010	128	126	28	75	25	20	60	46	Caucasian	1
20	Szendroi	2011	204	103	39	122	43	25	47	31	Caucasian	0.43
21	Safarinejad	2012	162	324	57	94	11	90	169	65	Caucasian	0.434
22	Jurecekova	2013	311	256	79	154	78	49	126	81	Caucasian	1
23	Lu	2015	352	352	94	191	67	97	175	80	Asian	0.95
24	Han	2017	244	222	48	102	94	34	96	92	Asian	0.313

### Meta-analysis results

*ESRα rs9340799 polymorphism and susceptibility to PC.* To assess the association of ESRα rs9340799 polymorphism with PC, 17 studies were included in this meta-analysis with 3960 PC cases and 4848 normal controls (Table 2). Both allele and additive models were tested in the overall population. PC susceptibility in Caucasian population showed strong association in the allele model (AvsG), and patients carrying mutant gene A were 0.84 times incidence rate of susceptibility to PC (OR = 0.84, 95 CI%: 0.72–0.96, *P* = 0.000). In the additive model (AAvsGG), PC case group obtained 0.75 times of probability on AA genotype and enhanced the risk of disease (OR = 0.75,95% CI, 0.56–1.00, *P* = 0.002). In contrast, there was no significant association between Asian and African populations in all the models. Detailed results were listed in Table 2. Test of heterogeneity in the other models (AAvsAG+GG, AA+AGvsGG, AGvsGG) were not significant among different races of populations.

**Table 2:** Meta-analysis of the association between ESRα rs9340799 and prostate cancer

ESRα rs9340799		AvsG (allele model)			AAvsGG (additive model)		
Population	*N*	OR (95% CI)	P_h_	P_OR_	OR (95% CI)	P_h_	P_OR_
Overall	17	0.84 (0.75–0.94)	0.002	0.001	0.77 (0.61–0.97)	0.038	0.011
Caucasian	11	0.84 (0.72–0.96)	0.015	0.000	0.75 (0.56–1)	0.076	0.002
Asian	4	0.92 (0.77–1.11)	0.410	0.493	0.96 (0.59–1.55)	0.874	0.736
African	2	0.65 (0.48–0.88)	0.015	0.800	0.49 (0.24–1)	0.049	0.788
**ESRα rs2234693**		**CvsT (allele model)**			**CCvsTT (additive model)**		
Population	*N*	OR (95%CI)	P_h_	P_OR_	OR(95%CI)	P_h_	P_OR_
Overall	24	1.09 (1.00–1.18)	0.037	0.000	1.21 (1.01–1.44)	0.030	0.000
Caucasian	13	1.11 (1.00–1.23)	0.070	0.004	1.26 (1.01–1.58)	0.062	0.001
Asian	9	1.02 (0.86–1.21)	0.571	0.004	1.05 (0.72–1.52)	0.517	0.003
African	2	1.24 (0.94–1.64)	0.134	0.636	1.52 (0.86–2.69)	0.143	0.669

OR odd ratio, 95% CI confidence interval, P_OR_ value for the test of association, P_h_ value for heterogeneity analysis.

**Figure 2:**
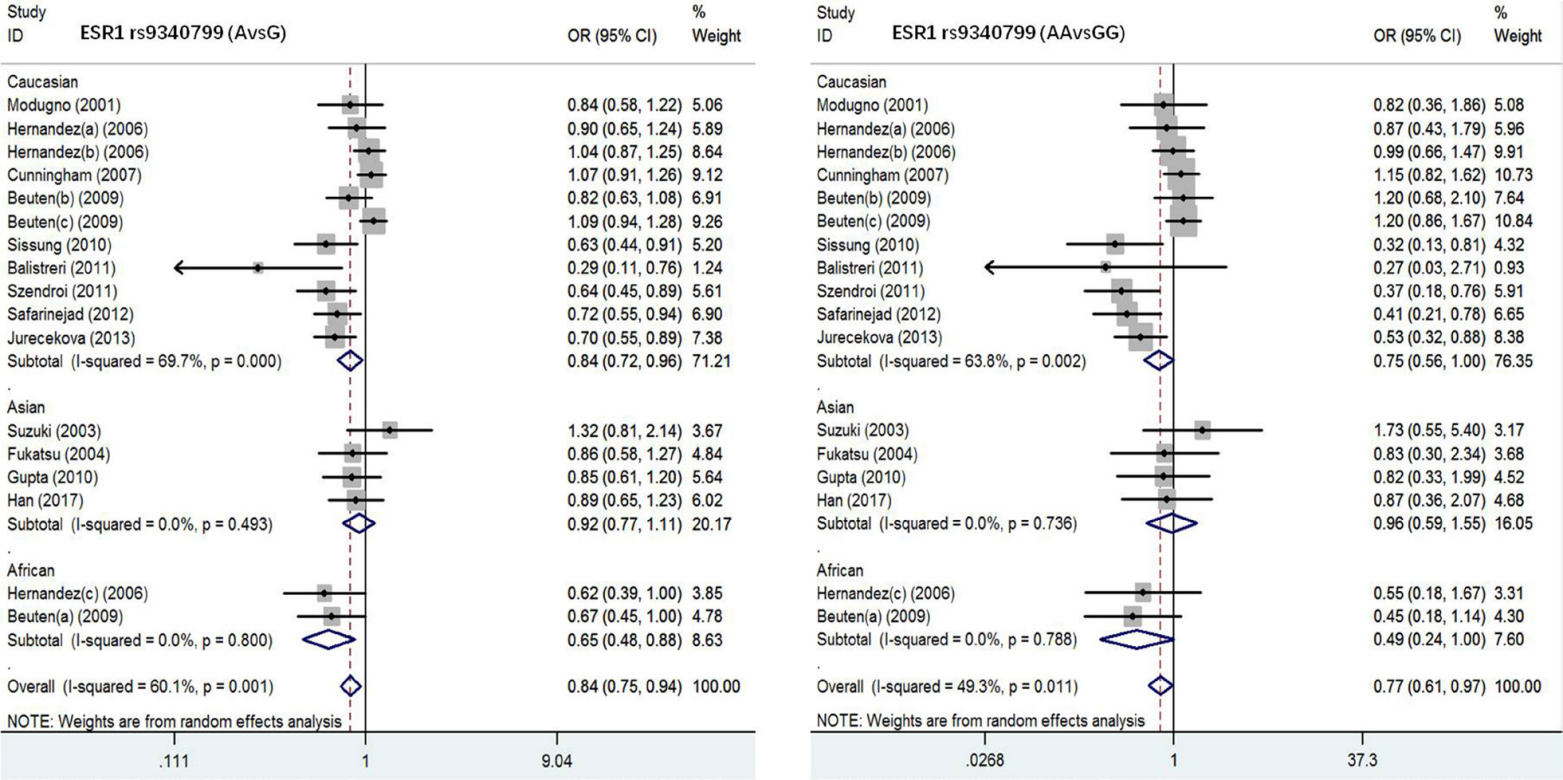
Forest plot of the association between ESR1 rs9340799 and prostate cancer risk(AvsG, AAvsGG)

**Figure 3:**
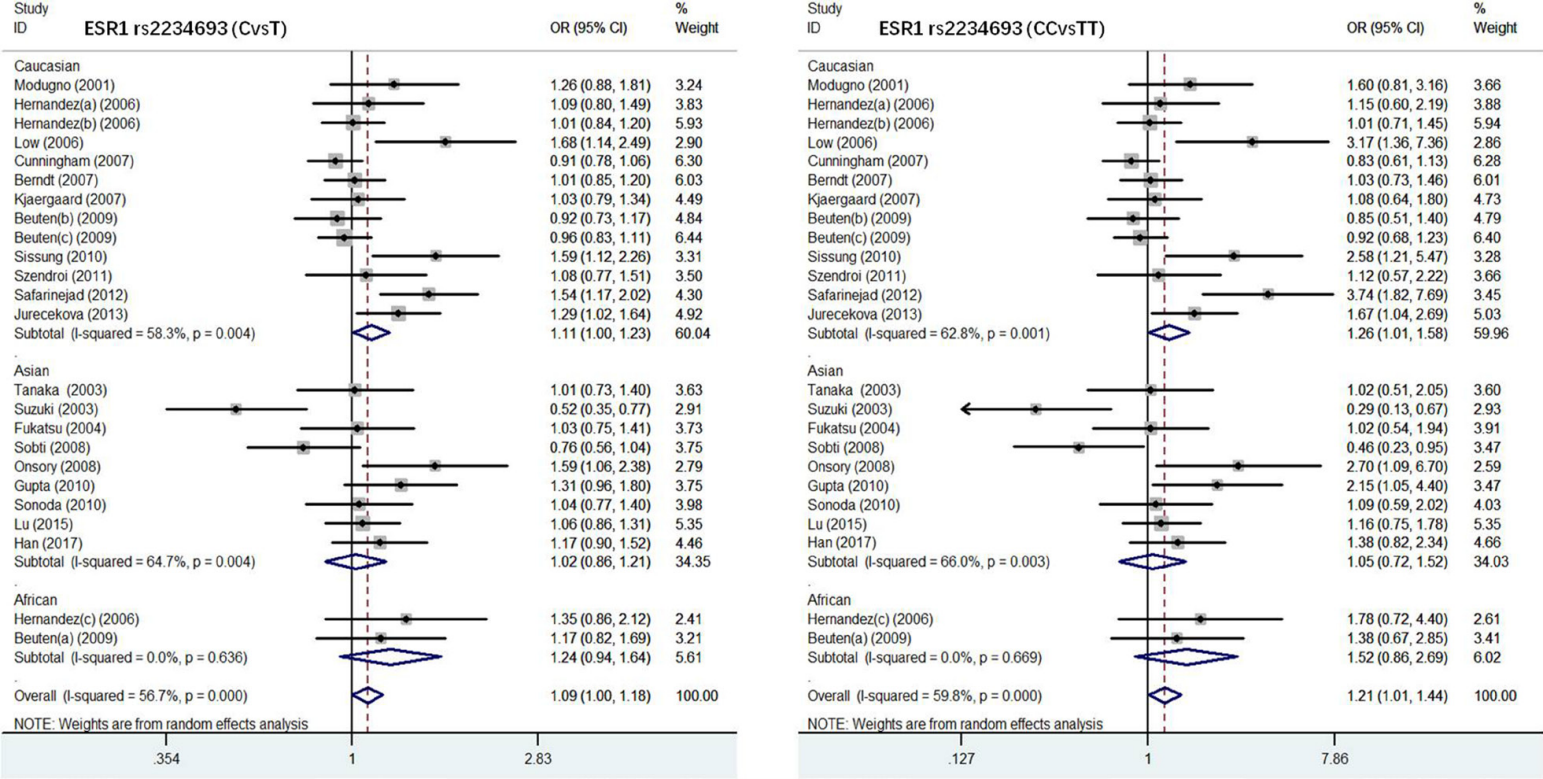
Forest plot of the association between ESR1 rs2234693 and prostate cancer risk(CvsT, CCvsTT)

*ESRα rs2234693 polymorphism and susceptibility to PC.* There were 24 studies with 5477 PC cases and 10708 normal controls in this meta-analysis to evaluate the association of ESRα rs2234693 polymorphism with PC (Table 2). In allele model, the risk genotype of wild type T was used to assess the relationship between C gene mutation and susceptibility to PC. Our results showed that there was statistical heterogeneity among these studies (I^2^ = 56.75, *P* = 0.003), so the random effects model was chosen for meta-analysis. As shown in Figure 2 and Figure 3, forest maps presented that PC patients carrying C genotype got a higher risk than people with T genotype (OR = 1.09, 95% CI:1.00–1.18, *P* = 0.000), especially in the European (OR = 1.11, 95% CI:1.00–1.23, *P* = 0.004) and Asian populations (OR = 1.02, 95% CI:0.86–1.21, *P* = 0.004), however, Africans were not correlated with either genotype (OR = 1.24, 95% CI:0.94–1.64, *P* = 0.636). In the dominant model, with TT genotype reference, mutation homozygous CC genotype was evaluated and discussed with PC susceptibility. Meta-analysis was conducted with a random effects model (I^2^ = 59.8, *P* = 0.001), CC genotype in overall population was more inclined to PC than people with TT genotype (OR = 1.21, 95% CI:1.01–1.44, *P* = 0.000). The result in different races was consistent with allele model that there was the remarkable relationship in European or Asian populations, but no correlation in African populations (Europeans, OR = 1.26, 95% CI:1.01–1.58, *P* = 0.001; Asians, OR = 1.05, 95% CI:0.72–1.52, *P* = 0.003; Africans, OR = 1.52, 95% CI:0.86–2.69, *P* = 0.669). Other models (CCvsCT+TT, CC+CTvsTT, CTvsTT) were not heterogeneous among different races, either.

### Allele frequency of *ESRα rs9340799, rs2234693* and comparing to the 1000 genome population

In Table 3, we demonstrated the distinct difference of allele frequencies in Caucasian, Asian and African populations in the meta-analysis of ESRα rs9340799 and rs2234693, which were consistent with the data of 1000 genome population project, European ancestry (EUR), Asian ancestry (EAS), African ancestry (AFR).

**Table 3:** The allele frequency comparison between the meta-analysis and 1000 genomes project

ESRα rs9340799	Meta-analysis (alleles frequencies)				1000 Genomes (alleles frequencies)	
Populations	Cases		Controls			
	A	G	A	G	A	G
Caucasian	0.620	0.380	0.647	0.353	0.308 EUR	0.692 EUR
Asian	0.767	0.233	0.785	0.215	0.194 EAS	0.806 EAS
African	0.655	0.345	0.741	0.259	0.265 AFR	0.735 AFR
All	0.644	0.356	0.677	0.323	0.281 ALL	0.719 ALL
**ESRα rs2234693**	**Meta-analysis (alleles frequencies)**				**1000 Genomes (alleles frequencies)**	
Populations	Cases		Controls			
	C	T	C	T	C	T
Caucasian	0.482	0.518	0.457	0.543	0.423 EUR	0.577 EUR
Asian	0.467	0.533	0.462	0.538	0.400 EAS	0.600 EAS
African	0.547	0.453	0.495	0.505	0.570 AFR	0.430 AFR
All	0.467	0.533	0.460	0.540	0.446 ALL	0.554 ALL

EUR European ancestry, EAS Asian ancestry, AFR African ancestry, ALL All individuals from phase 1 of the 1000 Genomes Project.

### Publication bias and sensitivity analysis

**Figure 4:**
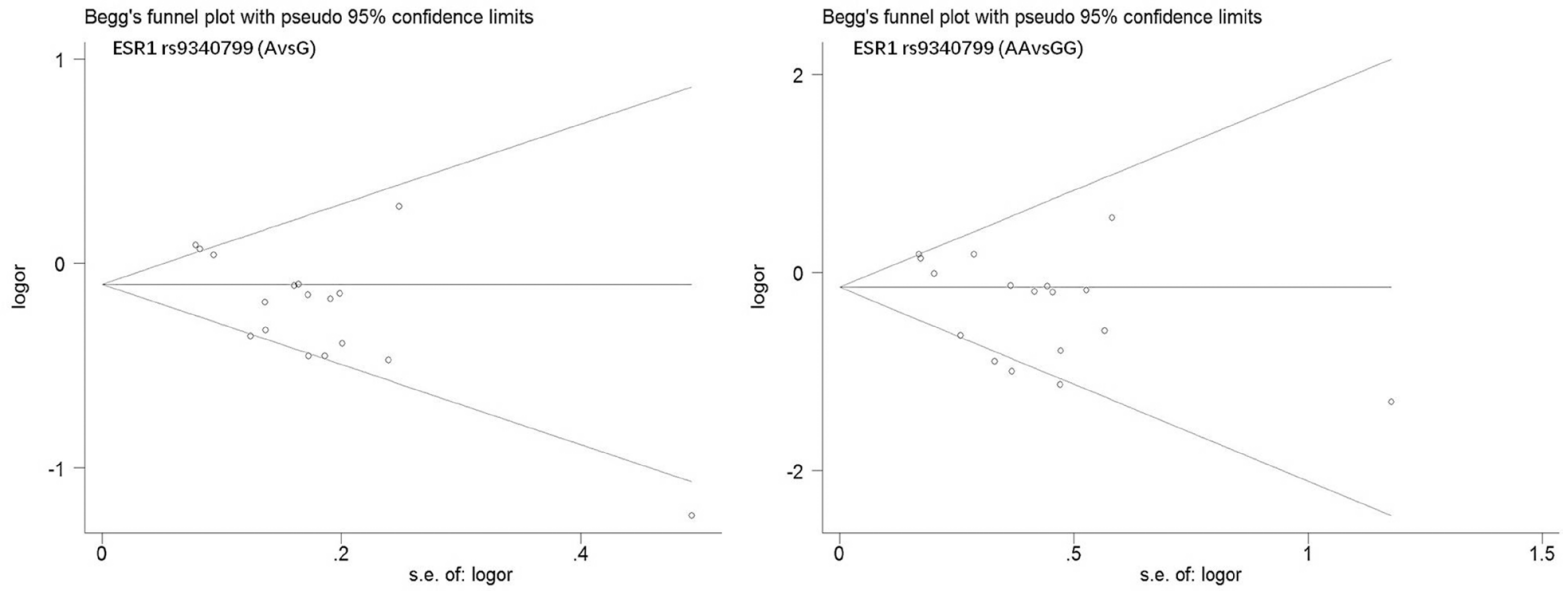
Begg's funnel plot of publication bias in meta-analysis of the association between ESR1 rs9340799 and prostate cancer risk(AvsG, AAvsGG)

**Figure 5:**
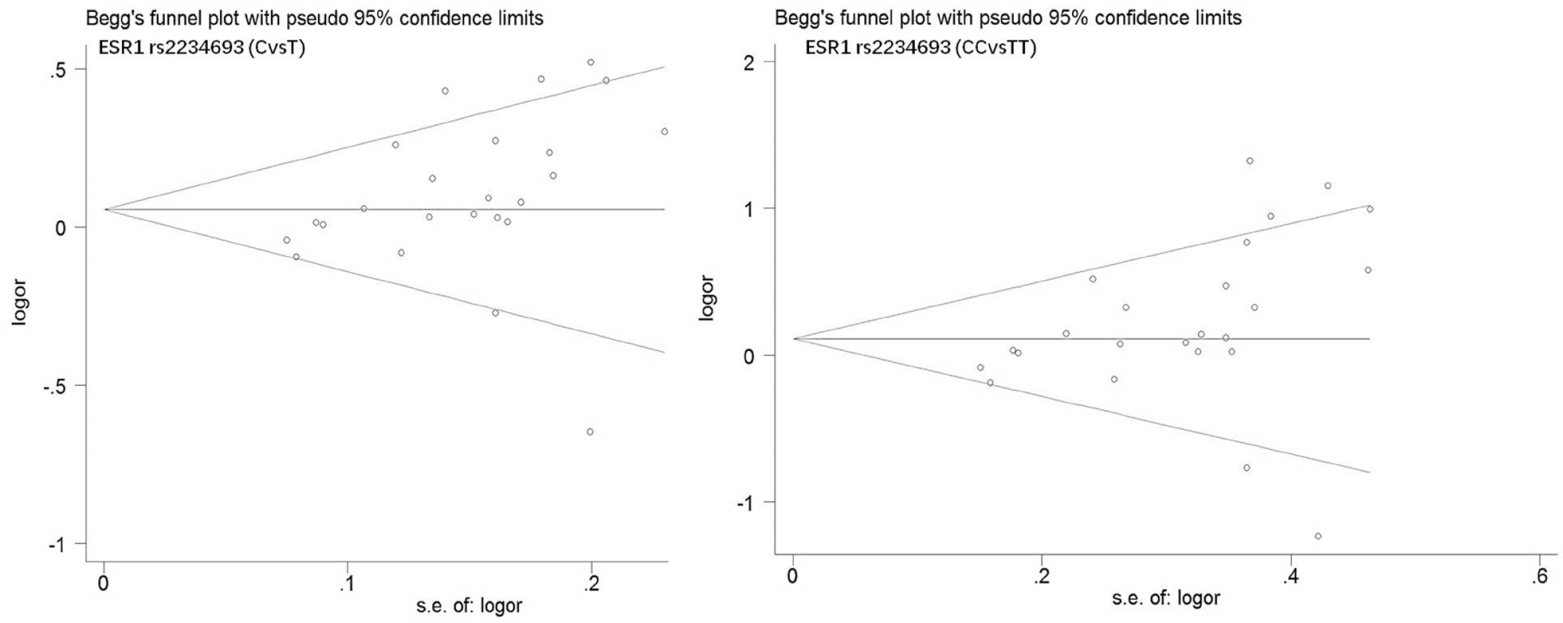
Begg's funnel plot of publication bias in meta-analysis of the association between ESR1 rs2234693 and prostate cancer risk(CvsT, CCvsTT)

**Figure 6:**
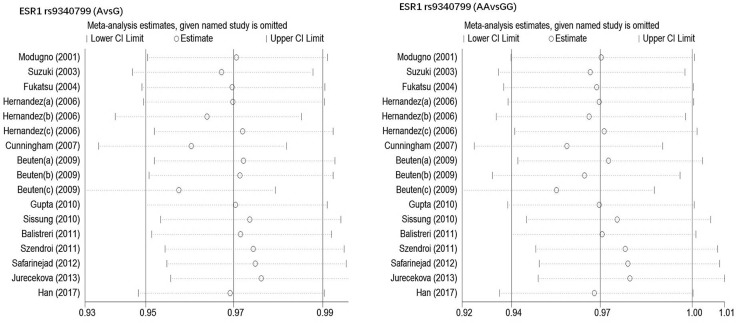
Sensitive analysis to assess the stability of meta-analysis between ESR1 rs9340799 and prostate cancer risk(AvsG, AAvsGG)

**Figure 7:**
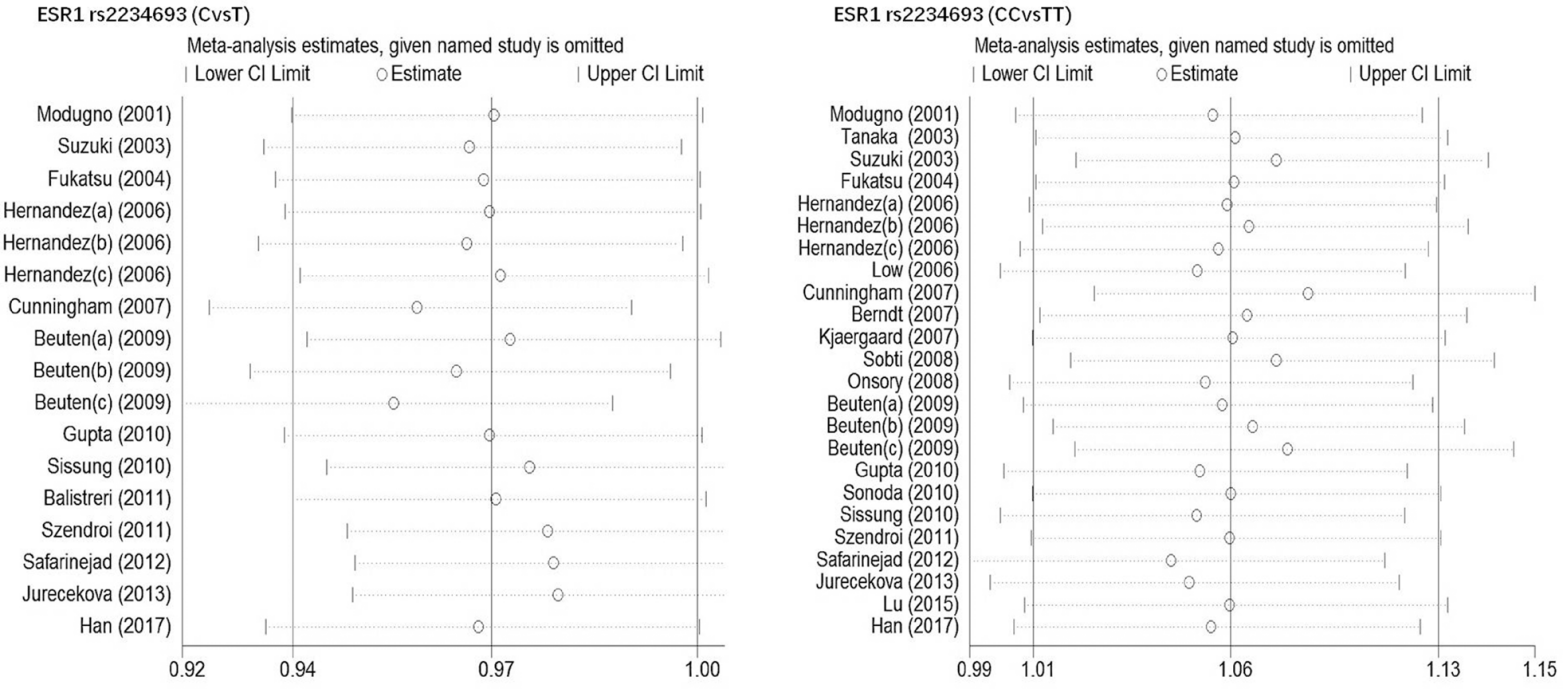
Sensitive analysis to assess the stability of meta-analysis between ESR1 rs2234693 and prostate cancer risk(CvsT, CCvsTT)

Begg's funnel plot and Egger's test were performed to estimate publication bias. As shown in Figures 4 and Figure 5, the funnel plots presented scattered distribution but large symmetry, indicating that there was no obvious evidence of publication bias in ESRα rs9340799 (AvsG *P* = 0.081, AAvsGG, *P* = 0.111) and ESRα rs2234693 (CvsT, *P* = 0.166, CCvsTT, *P* = 0.136). In addition, we conducted sensitivity analysis to assess the stability of meta-analysis between ESRα rs2234693 and PC risk. As shown in Figure 6 and Figure 7, there was no significant difference, suggesting that articles selected in this research were available.

## DISCUSSION

ESRα is a kind of ligand-activated nuclear transcription factor and mainly distributes at the clearance between the basal epithelial cells and the matrix of the prostate. The gene coded ESRα was located at 6q25.1 of the human chromosome, which possessed 140,000 bp and consisted of eight exons and seven introns. Two of the most common polymorphic sites in ESRα were rs9340799 and rs2234693, both of which were situated in the first intron with 50 bp of distance, the same as the positive chromosome area using large-scale genetic scanning chain analysis as reported previously [37]. The first intron lied at the end of amidogen, which was located in the main transcriptional domain, and contained the major adjustment sequences such as promoters and enhancers. Base T/C or A/G substitution in the area may cause the loss of existed restriction enzyme site and produce a new one instead, and each behavior may lead to the error splicing and bring abnormal expression products. In addition, estrogen wouldn't work without combining with ESRα, and the abnormal ESRα expression would directly induce the ultimate physiological effect of the estrogen in body, and finally affect the occurrence and development of PC. Therefore, it is likely that the genetic polymorphisms at ESRα rs9340799 and rs2234693 may contribute to the risk of PC.

So far there were four published papers [9–12] in this meta-analysis concerning the association of the polymorphisms of ESRα (rs9340799 or rs2234693) with PC susceptibility. However, the number of references regarding those results was unstable due to different publication period and standard for data screening. In this paper, 17 studies on ESRα rs9340799 and 24 studies on ESRα rs2234693 were included according to the strict inclusion and exclusion critiria, through searching in both Chinese and English databases. We found that there was statistically significant difference in distribution frequency of genetic polymorphisms of ESRα allelic genes between cases and controls (*p* < 0.05). Further analysis on PC patients from different races indicated that both base A and AA of ESRα rs9340799 may increase the risk of PC in overall population, particularly in European, however, the association was not significant in the populations of Asian and African. Similarly, a remarkable correlation was found in base C and CC of ESRα rs2234693 in Europeans and Asians, suggesting a higher risk of PC in these populations than that in Africans. Considering the limited samples from only two studies in African in this meta-analysis, studies with larger sample size including diverse ethnic populations are required to investigate the association between the polymorphisms of ESRα and the susceptibility of PC.

Consistent with the report of Gu [11], we observed that there was a certain correlation between PC and ESRα rs9340799, and the precision of such correlation reached to polymorphism of the site, which induced an affirmative result through further study on larger samples. Meanwhile, it should be noted that the control group of ESRα rs9340799 had no representativeness in the h-w genetic balance test according to the standard of *P* > 0.05, while it was still acceptable if the standard was *P* > 0.01. All results in this study were selected according to the standard of *P* > 0.05, therefore, larger sample size was required in the future. Besides, different conclusions were reached in this study on the correlation between the genetic sites of ESRα rs2234693 and PC compared with previous studies. On the one hand, as reported before, PC was a kind of disease affected by multiple genes, which induced various clinical features. It was inevitable to reach negative results if the samples were inadequate or assigned randomly.

It is worth mentioning that there were still have several limitations in this meta-analysis: 1) For lack of linguistic experience, databases used in this study were limited to English and Chinese; 2) This study focused on the analysis on ESRα rs9340799 and rs2234693 only, without considering the interactions between other genes and environmental factors. Therefore, stricter design was needed to control destructive factors, and larger samples and homogeneous cases were required in the comparable and prospective studies. At the same time, the interactions between the genes and environmental factors would be fully considered in the pathogenesis of PC, which provided more reliable evidences for basic study and clinical treatment.

Despite the limitations, this meta-analysis revealed that ESRα rs9340799 and rs2234693 were associated with susceptibility to PC, and studies with larger sample size are needed to define the association between the polymorphisms of ESRα rs9340799 and rs2234693 and the risk of PC in the future.

## MATERIALS AND METHODS

### The inclusion and exclusion criteria

Inclusion criteria: (1) the type of study was case-control; (2) cases were histologically diagnosed with PC patients, the pathological type was adenocarcinoma, control group was unrelated healthy people; (3) the distribution of genotypes was fully justified with Hardy-Weinberg genetic balance law; (4) the literature provided a complete genotype data. Exclusion criteria: (1) there was only case but without control group; (2) study which was repetitive publication; (3) incomplete data [13].

### Literature search

Keywords “single nucleotide polymorphism or SNP or variants”, “prostate cancer or carcinoma”, “estrogen receptor alpha or ESR1”, were searched in PubMed, Embase, Cochrane Library, China Biology Medicine (CBM), China science and technology journal database (VIP), Chinese national knowledge infrastructure (CNKI) and Wanfang data knowledge service platform, from its inception to May 2017.

### Quality assessment and data selection

Two researchers independently read abstracts, and the eligible literatures were intensively read for further study. Any differences were settled by discussion or the third researcher who decided whether be taken into the following. Quality of included reference were evaluated with Cochrane Reviewer ‹s Handbook 5 System Evaluation Handbook: (1) the diagnostic criteria was clear; (2) the sample size was sufficient; (3) the case and control groups were comparable; (4) statistical analysis method was appropriate; (5) existence of bias was discussed. Each term above was recorded 1 points, > 2 points for reliable quality. The following data was extracted from each included literature: the first author, year of publication, sample size, race, genotype frequency of case and control group, Handy-Weinberg.

### Statistical processing

Meta-analysis was performed with Stata software. *Q*-test and I^2^-test were firstly used to judge the heterogeneity. *Q*-test *P* > 0.1 or I^2^ < 50% indicated that no significant heterogeneity was found between these studies, instead, fixed effect model should be used to combine. On the contrary, *Q*-test *P* < 0.1 or I^2^ > 50% stood for heterogeneity between these results, and such heterogeneity was not found from clinic, therefore, these data was merged using a random effect model. The combined odds ratio (OR) and 95% confidence intervals (CI) were then calculated, and difference (*P* < 0.05) was regarded as statistical significance. Egger's and Begg ‘s test was applied to assess the publication bias. At last, OR value distribution was demonstrated with forest map, funnel plots and sensitive graphs, respectively.
